# Creating a medical education enterprise: leveling the playing fields of medical education vs. medical science research within core missions

**DOI:** 10.1080/10872981.2017.1377038

**Published:** 2017-09-20

**Authors:** Satid Thammasitboon, B. Lee Ligon, Geeta Singhal, Gordon E. Schutze, Teri L. Turner

**Affiliations:** ^a^ Center for Research, Innovation and Scholarship in Medical Education, Department of Pediatrics, Baylor College of Medicine, Houston, Texas, USA; ^b^ Department of Pediatrics, Baylor College of Medicine, Houston, Texas, USA

**Keywords:** Scholarship, faculty development, clinician-educator, organization

## Abstract

**Background**: Unlike publications of medical science research that are more readily rewarded, clinician-educators’ scholarly achievements are more nebulous and under-recognized.

**Objective:**Create an education enterprise that empowers clinician-educators to engage in a broad range of scholarly activities and produce educational scholarship using strategic approaches to level the playing fields within an organization.

**Design:** The authors analyzed the advantages and disadvantages experienced by medical science researchers vs. clinician educators using Bolman and Deal’s (B&D) four frames of organization (structural, human resource, political, symbolic). The authors then identified organizational approaches and activities that align with each B&D frame and proposed practical strategies to empower clinician-educators in their scholarly endeavors.

**Results**: Our medical education enterprise enhanced the *structural* frame by creating a decentralized medical education unit, incorporated the *human resource* component with an endowed chair to support faculty development, leveraged the *political* model by providing grant supports and expanding venues for scholarship, and enhanced the *symbolic* frame by endorsing the value of education and public recognition from leaderships. In five years, we saw an increased number of faculty interested in becoming clinician-educators, had an increased number of faculty winning Educational Awards for Excellence and delivering conference presentations, and received 12 of the 15 college-wide awards for educational scholarship. These satisfactory trends reflect early success of our educational enterprise.

**Conclusions**: B&D’s organizational frames can be used to identify strategies for addressing the pressing need to promote and recognize clinician-educators’ scholarship. We realize that our situation is unique in several respects, but this approach is flexible within an institution and transferable to any other institution and its medical education program.

**Abbreviations**: B&D: Bolman and Deal; CRIS: Center for Research, Innovation, and Scholarship; OOR: Office of Research

## Introduction: recognizing educational scholarship

Most academicians are painfully aware of the old adage ‘Publish or Perish,’ a trite summation of the pressure many faculty members encounter as they seek advancement, recognition, and promotion [1]. Medical educators face even greater challenges, as many of their scholarly achievements have not always received proper recognition, often are more nebulous than are standard publications, and require different criteria for appreciation and positive evaluations [2–4]. As Mensah noted almost 35 years ago, the process of granting tenure and promotion at many academic institutions relies on measuring only the rate of grant applications and the quota of publications [5]. Despite well-intentioned efforts to recognize equally the areas of medical research, clinical efforts, and educational scholarship [6–8], as well as the Association of American Medical Colleges’ call for institutional adoption of five specific criteria to evaluate faculty seeking academic promotion as educators [9], the reality is that research, with its ability to demonstrate financial gains, is more easily – and, hence, usually more readily – recognized and rewarded. Faculty promotions frequently follow suit [9]. In our department, we are fortunate to have a Chair who values all areas of effort and reminds the faculty that, *‘A commitment to excellence in all of our educational endeavors is central – to who we are, to how we are perceived by our community and colleagues, and to everything we hope to accomplish on behalf of the children and families we serve.’* Accordingly, we have created and developed a sustained educational enterprise that nurtures medical educators’ scholarly development, raises awareness of the value of their contributions, and properly recognizes their efforts, with the result that their scholarly achievements have increased, enhancing the status of medical education within the organization. We describe herein how the enterprise was founded and developed, using Bolman and Deal’s categories of management theories [10], and explain how integrating similar programs into the academic landscape can change the future of medical education by properly recognizing medical educators’ scholarly efforts.

## The charge: creation of a clinician-educator enterprise

As the largest department of pediatrics in the nation, we are widely heralded for our exceptional patient care and clinical research, with the unintended result that medical education historically took the proverbial but unwarranted ‘back seat’ and our medical educators faced formidable challenges in gaining promotions and tenure. Our Chair, recognizing the discrepancy and its inherent imbalance in scholarly recognition, charged us with establishing an enterprise that would highlight the scholarly achievements of our medical educators, foster robust educational scholarship, and facilitate the growth of young and mid-career faculty members. To those ends, we created and continue to expand a large enterprise that includes the formation of a Center for Research, Innovation and Scholarship (CRIS) grounded in Boyer’s criteria for identifying and implementing four areas of scholarly recognition: discovery, integration, application, and teaching [11,12]. These four areas are the cornerstones of CRIS’s efforts and emphases. The leadership then recruited faculty members with various areas of specialized expertise to form a multidisciplinary Core Faculty. CRIS initially provided technical assistance for statistics, research methodology, and medical writing. Subsequently, a competitive grant process and seed funding were established to support endeavors across the continuum of medical education. Under the direction of a Vice Chair of Education, CRIS functions as the ‘hub’ of the larger endeavor, the overall medical education enterprise under the direction of the Executive Vice Chair of Education.

## The challenge: empowering medical educators’ scholarship

Once CRIS was in place, we faced the daunting challenge of identifying areas we could address to raise the status of medical educational scholarship to that of basic science research. Scholarship has been defined as the public dissemination of efforts, whereby they can be subjected to peer review and critique and can provide a means to be reproduced and expanded by other scholars [13]. Over the years, we had invested significant time and effort to support educational scholarship and had addressed issues pertaining to the challenge. To move our education enterprise forward, it was critical for us to understand and systematically describe our past tasks, successes, and lessons learned. A better understanding about our education enterprise would guide our future efforts toward addressing the challenges ahead.

After performing an extensive literature search and holding consultations with academic and business leaders, we determined that the most informed framework is that provided by Bolman and Deal, which summarizes characteristics of various management theories into four categories or perspectives that they call ‘frames’ [10]. This model has been used effectively by researchers in various disciplines, including organizational management, educational leadership, and academic planning for diversity [14].

For each of the four frames (structural, human resource, political, symbolic), Bolman and Deal provide an organizational metaphor, describe central concepts, identify leadership styles, and delineate the basic leadership challenge. An important concept of this approach is that, as the authors point out, each frame has its own image of reality and a good organization will know how to reframe situations until they understand them adequately. Hence, the approach has considerable flexibility, which we found useful for laying the groundwork of our enterprise. In other cases, the four frames have emerged over the course of time [14].

These four frames provided us with the needed framework to define: (1) the advantages experienced by medical science research; (2) the pros and cons of engaging in educational scholarship; and (3) the strategies needed to empower the medical educator in scholarly endeavors. We describe herein how our strategies to empower medical educators’ scholarship are aligned with each frame with regard to its characteristics (Table 1).

**Table 1.** The Bowman and Deal’s frames of organization theories[10] and strategies we used to empower medical educators’ scholarly pursuits.

Frame	Definitions/descriptions	Prerequisites for success in medical science research	Our strategies to empower medical educators’ scholarly pursuits
Structural	Refers to the administrative structure frame inherent in an organization, as manifested in organizational charts, rules and policies, and goals and tasks, as well as infrastructures	Well-structured chain of duty/command within the Office of Research (OOR) Established paths to internal and external grant supports Established paths for promotion as researchers More outlets for journal publication	Delineated a clear and de-centralized organization (Executive Vice-Chair, Vice-Chair, Associate Vice-Chair for Educational Affairs) to promote collaboration across the educational enterprise Created a Center for Research, Innovation and Scholarship (CRIS) specifically for Medical Education Offered Educational Scholarship Grants to foster educational activities with potentials for scholarship Created a clear path for promotion as clinician-educators Broadly defined success as dissemination across various venues (publication, presentation, enduring materials)
Human Resource	Refers to how an organization optimizes the performance of members through recruiting personnel with proper expertise, supporting their needs, and developing and empowering its members	Availability of designated, experienced personnel (statistical, administrative support) within OOR Collaborative team bound by financial supports or clear job descriptions Mandatory protected time for research activities	Consolidated expertise within the department (CRIS) to support less experience medical educators Allocated financial supports for Endowed Chair in Medical Education to provide ample opportunities for faculty development Offered appropriate incentives (e.g., sponsoring travel) to level the playing field Supported community of medical educators within educational services to enhance scholarly collaboration
Political	Refers to the alignment of power and authority to acquire and manage resources, negotiate conflicts and form alliances needed for the organization to succeed	Research units aimed to achieve clear common goals Financial benefits to gain authority and power Quantity and quality of research publication as an objective measure for promotion portfolio.	Educated medical educators to capitalize scholarly products from educational services Educated medical educators about dissemination venues for different forms of scholarship Incorporate educational scholarship into an incentive program Expanded venues for faculty to engage in scholarly activities within the organization at local, regional and national levels (e.g., AAP Institutional membership). Incorporated criterion-based, self-nominated educational awards recognizing all forms of scholarly activities in medical education as an objective measure for promotion portfolio
Symbolic	Refers to the objects, events, people, or stories used to communicate missions and values, establish a cultural identify, and follow a shared vision	Critical components to the core mission Redefined perception of ‘true’ scientific inquiry and discovery Conventionally equate prestige and reputation	Provided authentic value with consistent messages from the top regarding educational excellence Reminded leaders at all levels about importance of educational mission and scholarship Provided opportunities for recognizing all forms of scholarship (research or non-research) equally

### Structural

The structural frame, which emphasizes goals, roles, and relationships and is commonly depicted on extensive organization charts with allocation of responsibilities, is focused on rules and procedures. Given its nature, medical educational scholarship may be bereft of a clear or supporting structure, and the conventional office of research may not support it. The broad range of scholarly activities in medical education renders the determination of their merits or success challenging. Further, when educational endeavors are implemented, outlets for dissemination of that scholarship, especially publications, are relatively limited. Medical educators’ efforts to identify structures that foster success in educational scholarship and the contextual elements for enabling the attainment of that success are ongoing [15].

#### Our strategies

Our first step in establishing this portion of the framework was to determine the roles that CRIS would play as a distinct decentralized organization under the leadership of the department’s Vice-Chairs for Educational Affairs. The decentralized organizational structure enhances efficiency and creativity, promotes collaboration for scholarship across the educational enterprise, and provides a track to enhance promotion. Subsequently, Medical Educational Scholarship Grants were created to foster educational activities that align with our educational mission and have potential for scholarship. These grants are divided into two categories: large funding (>$15k to $40k) and small funding (≤$15K). We usually grant as many as three large and six small grants annually. CRIS was designated as the appropriate entity for handling many of the administrative logistics of these grants, including sending out a call for submissions, evaluating the submissions, and accepting/rejecting the submissions, with accompanying funding. To ensure institution-wide cooperation, the medical educational enterprise’s leadership engages in ongoing communication with the local Institutional Review Board to educate its committee members about unique issues in educational scholarship versus other forms of research. Venues for publication and other forms of exposure, such as an educational newsletter and online repository (e.g., MedEdPortal), are sought to expand opportunities for dissemination of scholarship.

### Human resource

The human resource frame draws from psychology in its focus on relationships between organizations and people, with some theories using the extended family as a metaphor. The key strategy is to tailor the organization to address feelings, prejudices, skills, limitations, and other human needs in a fashion that encourages the employees. Efforts directed at helping employees feel good about themselves and the jobs they are doing are thought to encourage more productivity. Whereas individuals engaged in basic research usually are part of a research team recognized by the office of research and have expert support (e.g., statisticians and administrative personnel), medical educators’ teams often must work their efforts around teaching and clinical responsibilities, and they rarely have grant support. With no formal protected time, medical educators within a team must negotiate time and effort to collaborate on delivering services and acquiring the requisite scholarly skills to produce scholarship.

#### Our strategies

Many of the strategies we have implemented in creating and expanding the overall medical education enterprise are, admittedly, related directly to the size and financial resources of this particular department. For instance, we created a $2 million Martin I. Lorin Endowed Chair in Medical Education, which funds educational projects, supports individuals receiving an M.Ed., and helps provide salary support for staff and faculty involved in educational work. It also funds an annual Grand Rounds presentation given by an outside, invited speaker. Obviously, not all institutions will have the resources to establish such a Chair, but some other form of clear recognition in the form of a position and/or title could be implemented to raise awareness of the importance of medical education. We also provide annual funding for one mid-career and one junior faculty member to attend a national faculty development program and funding of a local Educational Scholars Fellowship program. The other steps we have taken are more easily incorporated into most programs. For instance, we provide encouragement in the form of appropriate incentives such as protected time for educational scholarly efforts and financial support for attending meetings. The department also recognizes medical educators with specified annual awards, such as Best Grand Rounds, Best Education Innovations, Best Mentor, and so forth, which benefit the recipients but do not pose a financial burden on the department. These awards are presented by the Department Chair at faculty meetings.

To encourage and expand medical educators’ expertise, we present an annual department orientation specifically for clinician-educators, an annual faculty retreat in education with specific sessions for networking, and a quarterly educational scholarship series, in addition to a Visiting Professorship and a Grand Rounds lectureship. By consolidating much of this effort in CRIS, we have been able to support and enhance the teaching and learning environments with workshops and retreats. We provide on-site faculty development (e.g., workshops and presentations on time management, statistics, and writing for publication), in addition to the >100 hours offered annually by the institution.

### Political

The political model or frame is rooted in the work of political scientists. Organizations are likened to arenas, contests, or even jungles in which different factors compete for power and scarce resources, and conflict is rampant. Characterized by bargaining, negotiation, coercion, and compromise, this frame has coalitions formed around temporal interests and issues. Problems often occur when power is centralized in the wrong place(s) or so dispersed that nothing gets done. Educational scholarship may suffer under this model due to its lack of clarity of terminology (i.e., ‘educational research’ vs. ‘educational scholarship’) [16], resulting in confusion and competing interests. The frequent involvement of large collaborations of stakeholders with several levels of expertise and interests may result in compromised efficiency. Competing interests (e.g., research vs. teaching vs. service) pose a challenge to promoting innovation in education and encouraging scholarship. Further, the dissemination of scholarship has its own challenges, with only a small subset of journals that accept descriptive/innovation articles and journal reviewers setting high expectations (80/20) [17,18].

#### Our strategies

The challenges that surround this frame have been the most difficult for us to address, as would likely be the case in other institutions. The competitive nature inherent in comparisons can be overcome by systematically awarding educational scholarship in various venues, beyond merely the recognition of publications and grant funding. We encourage our faculty to take advantage of the American Academy of Pediatrics institutional membership, which offers opportunities for regional and national involvement in scholarly activities within the academy. We also encourage faculty to explore and apply for regional and national leadership roles so that they not only raise awareness of the value of medical education but also have a voice in its growth and development. An important strategy has been to encourage medical educators to apply for institutional education awards (e.g., Fulbright & Jaworski, L.L.P Awards for Faculty Excellence), for which CRIS provides counsel and support. Also, the department recognizes recipients of education awards in different venues, including faculty meetings and the department quarterly magazine. As the enterprise has grown, we have sought to enhance CRIS’s influence by attracting and retaining top educators who have expertise in various fields. We have successfully added both core and adjunct faculty from other disciplines and institutions, who bring a wide spectrum of insights to the monthly meetings.

### Symbolic

Drawing from social and cultural anthropology, these theories treat organizations as a culture with rituals, ceremonies, stories, heroes, and myths rather than rules, policies, or managerial authority. Problems arise when the members fail to play their roles well, the symbols lose their meanings, or ceremonies and rituals are diminished. In this context, educational excellence impacts the institution’s reputation or image. Educational scholarship often suffers from not being as valued as is basic research, especially when it is considered a ‘routine’ or ‘expected’ academic duty or merely a by-product of teaching and learning. The notion that educational scholarship is simply ‘soft science’ needs to be replaced with appreciation for the time and expertise that are required for providing genuine scholarship, and the appropriate recognition of faculty members’ efforts accordingly.

#### Our strategies

In our department, medical education scholarship receives equal acknowledgement with other achievements at faculty meetings, where it is recognized in the various categories of growth and development. To ensure that appreciation for educational scholarship ‘filters down,’ Section Chiefs receive from the Vice Chair for Education periodic updates and education about the importance of the educational mission. Recognizing the tendencies to underrate educational scholarship, we implemented a 3-M faculty evaluation and incentive program. With the support of our Chair, increased emphasis was placed on medical education in the process of evaluating faculty members for incentives. We further established department Teaching Awards and Awards for Educational Excellence within sections of the department. These awards recognize faculty for their individual contributions and are presented in large faculty meetings, with additional recognition given in department publications and correspondence. Educational workshops and educational enduring materials are recognized fully as scholarship, and faculty receive travel support to present their educational scholarship.

## The endeavor: harvesting early success

**Figure 1.**
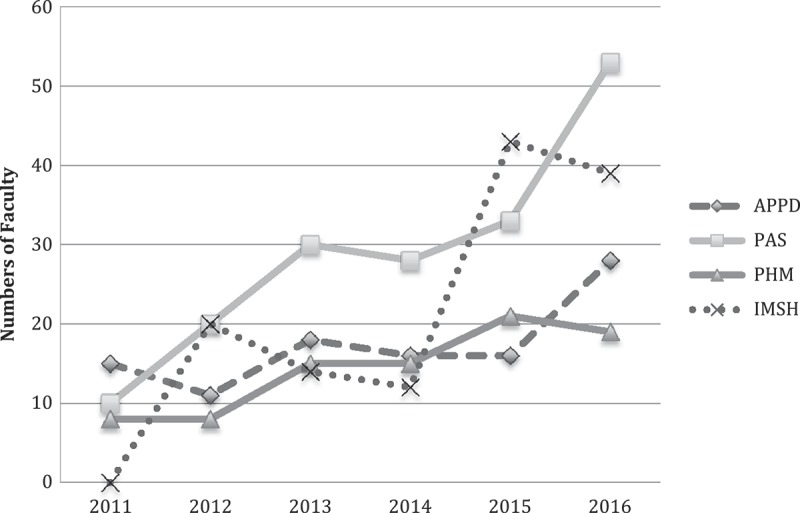
An increase in numbers of faculty delivering presentations (i.e., abstracts, workshops, plenary sessions, special interest group sessions) at selected medical education conferences (APPD - Association of Pediatric Program Directors, PAS - Pediatric Academic Societies, PHM - Pediatric Hospital Medicine, IMSH - International Meeting on Simulation in Healthcare).

**Figure 2.**
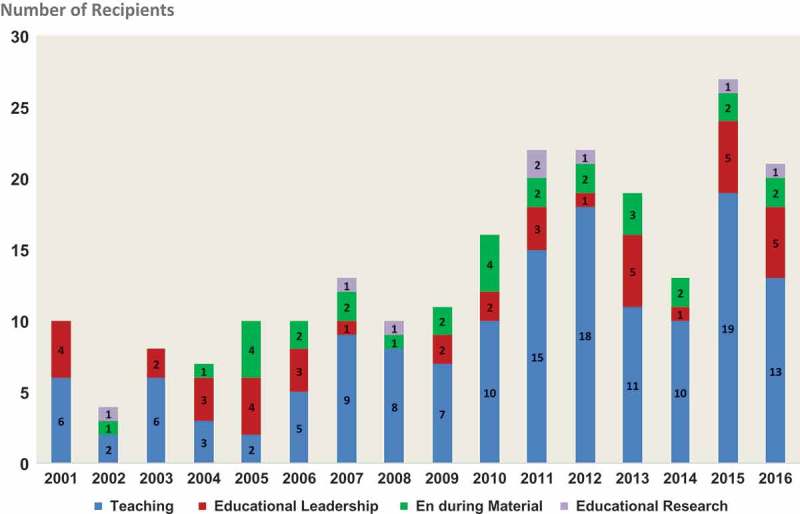
Numbers of Fulbright and Jaworski Award recipients in different categories over time.

The department has witnessed a trajectory of improvements in several areas of medical education during the recent years. We have seen an increased number of faculty interested in becoming clinician-educators energized to engage in scholarly activities in medical education. During the past five years, our faculty have received 12 of the 15 college-wide awards for educational scholarship. The number of faculty delivering conference presentations (i.e., abstract presentations, workshops, and platform presentations) has been increasing steadily (Figure 1). The number of Fulbright & Jaworski Educational Awards for Excellence given by our institution also has been increasing (Figure 2). These satisfactory trends not only reflect early success of our educational enterprise but also indicate ongoing endeavor to enhance the status of medical education within the organization.

To capitalize on this heightened energy and effort to excel in medical education, we identified areas for continued improvement. We strategically expanded CRIS’s structure by appointing a junior faculty member as a CRIS Scholar-in-Residence. We also established a collaboration with a local university and recruited experts to serve as adjunct faculty. To optimize the return (i.e., scholarship) from our investment, we added additional faculty-development series focused on developing skills pertaining to educational scholarship.

## Conclusion

We present here how we incorporated the four categories of management theory, as articulated by Bowman and Deal, into various components of developing a sustained education enterprise to nurture medical educators’ scholarly development. We recognize that our situation is unique in several respects, but the approach we used is transferable to any other institution and its medical education program. The value of this approach is twofold: (1) it offers different ways to evaluate an institution’s needs and provide changes and academic support to meet those needs, and (2) it is an incredibly flexible instrument that can be routinely adjusted to meet the particular needs of an institution and, in turn, each component can be modified for each component of the scholarly enterprise.
